# Seroprevalence of zoonotic abortive diseases and their associated risk factors in Tunisian sheep

**DOI:** 10.1186/s12917-022-03541-9

**Published:** 2023-02-15

**Authors:** Kaouther Guesmi, Sana Kalthoum, Aymen Mamlouk, Mohamed Naceur Baccar, Bassem BelHajMohamed, Haikel Hajlaoui, Aymen Toumi, Jamel Cherni, Chédia Seghaier, Lilia Messadi

**Affiliations:** 1 National Center for Zoosanitary Vigilance, Ministry of Agriculture, Water Resources and Fisheries, 38 Avenue Charles Nicolle, 1082 Tunis, Tunisia; 2 Department of Microbiology and Immunology, Institution of Agricultural Research and Higher Education, National School of Veterinary Medicine, Univ. Manouba, 2020 Sidi Thabet, Tunisia; 3 State-owned Land Office, Ministry of Agriculture, Water Resources and Fisheries, Tunis, Tunisia

**Keywords:** Abortion, Seroprevalence, ELISA, Brucella spp, Coxiella burnetii, Toxoplasma gondii, Sheep, Risk factors, Tunisia

## Abstract

**Background:**

Abortion is a serious problem for sheep flocks and it is responsible for considerable economic losses. The epidemiological situation of abortion causing agents in sheep is poorly documented in Tunisia. This study aims to investigate the status of three abortion causing agents (*Brucella spp, Toxoplasma gondii, and Coxiella burnetii*) among organized flocks in Tunisia.

**Results:**

A total of 793 sample blood collected from twenty-six flocks in seven governorates in Tunisia, were tested by indirect enzyme-linked immunosorbent assay (i-ELISA) for antibodies against three abortion causing agents (*Brucella spp*, *Toxoplasma* *gondii,* and Coxiella burnetii). Risk factors for individual-level seroprevalence were analyzed using a logistic regression model. Results revealed that 19.7%, 17.2%, and 16.1% of the tested sera were positive for toxoplasmosis, Q fever, and brucellosis, respectively. Mixed infection was found in all the flocks with 3 to 5 responsible abortive agents simultaneously.

Logistic regression showed that the management practices (control of new introduction, common grazing and watering point, workers exchange, presence of lambing box on the farm) and the history of infertility and the presence of abortion in neighboring flocks were likely to increase the probability of being infected by the three abortive agents.

**Conclusions:**

Evidence of the positive relationship between seroprevalence of abortion causing agents and several risk factors, suggests further investigations to better understand the etiology of infectious abortions in flocks to develop an applicable preventive and control program.

## Background

Sheep farming plays an important role in food security and food self-sufficiency. It represents the main source of income for the rural population of Tunisia. It contributes to around 40% of red meat production (Ibidhi et al., [1], GIVLAIT, [2]). However, this sector has experienced several challenges in the last decades due to climatic, economic, social, environmental, and animal health factors (Ben Salem et al., [3]). It was demonstrated that the health status of livestock is the most important factor influencing productivity (Lamy et al. [4]). Abortions are one of the major problems of sheep farming that cause considerable economic losses (infertility, stillbirth, decrease in production, treatments costs, etc.). Non-infectious and infectious agents are responsible for abortion but the infectious agent is the most implicated (Givens et al. [5], Holler [6]). Zoonotic potency of several abortion causing agents such as *Brucella spp., Coxiella burnetii, Toxoplasma gondii*, etc. was described. In Tunisia, abortion is a preoccupancy with health problems for farmers and humans. Previous studies showed that many abortion causing agents in sheep in Tunisia, such as *Brucella* *spp, Toxoplasma gondii, Coxiella burnetii*, and *Chlamydia* *abortus*, were endemic (Rekik et al. [7], Khammasi et al. [8], Barkallah et al. [9], Elandoulsi et al.  [10]). Brucellosis remains one of the most worrying zoonosis in Tunisia and the incidence in humans varied between 2.9 per 100,000 inhabitants in 2008 and 3.9 per 100,000 inhabitants in 2015 (Khbou et al. [11]). Studies on Toxoplasmosis in humans in Tunisia revealed a high seroprevalence rate which is estimated at 58.4% in the North-West of the country (Bouratbine A et al., [12]). Other studies carried out on pregnant women showed that seroprevalence rates ranged between 39.3% and 47.4% (Sellami et al., 2010 [13]; Fakhfakh et al., 2013 [14]). In sheep, previous surveys reported an infection rate of 73.6% of toxoplasma gondii using a modified agglutination test (MAT) (Gharbi M, & et al. [15], M. Rouatbi et al. 2019 [16]). Using molecular tools, Boughattas et al. (2014) [17] observed the highest infection rate in ewe tissues (50%) in Tunis City. Other studies determined the molecular prevalence of *T. gondii* in samples collected from sheep, with infection rates ranging between 5.7% and 25.5% (Gharbi et al. [15], Khayeche et al., 2013 [18]). Q fever is also considered an important abortion disease and studies that investigated infection in animals showed a variation in seroprevalence between areas. So, the lowest value of the seroprevalence was found in the survey conducted in 2005 where 7% of sheep without a history of abortions were seropositive for *C. burnetii* against 12% of sheep with a history of abortions (Rekik et al. [7]). According to Berri study, 19% of the sampled sheep were positive for *C. burnetii* using PCR (Berri et al. [19]). More recently, a survey carried out in central-east of Tunisia on sheep using PCR and ELISA revealed a prevalence of 11.8% (Barkallah et al. [9]). In humans, the seroprevalence seems to be similar to that found in animals (Omezzine Letaief et al. [20] Kaabia et al. [21]). As Known, many factors may play an important role in the introduction, the spread, and the persistence of these abortion diseases among animals and herds such as the herd management (reproduction, watering, crazing…) and the individual factors like the breed, age, sex….(Alemayehu et al. [22]).Therefore, the risk of introduction and spread of these diseases is amplified by uncontrolled livestock movements that occur in neighboring countries especially during religious (Eid al-Adha, Ramadan, and Hadj) and social events (weddings, etc.) (Bouguedour et al. 2016 [23]).

Despite the previous studies that demonstrated the presence of these diseases among sheep, the prevalence of the abortion causing agents in sheep farms remained largely unknown at the national level in Tunisia. In addition, the proportion of undiagnosed abortions is significant and is probably due to the presence of non-infectious causes, which are difficult to detect (Van Engelen et al. [24]). Potential risk factors associated with the abortion disease were poorly documented.

An effective system of surveillance and control measures requires the updating of data about these diseases, including their true prevalence in sheep, and investigating their related risk factors. In this study, we investigate the seroprevalence of three abortion causing agents (*Brucella* *spp, Toxoplasma gondii,* and *Coxiella burnetii*) in sheep and the risk factors associated with the persistence of these pathogens in the farms. Our study targeted organized farms because they include most state farms where animals are bred for genetic improvement and they supply other farms with young females and males. This category represents about 20% of the population of sheep and goats in Tunisia where registers of breeding were available (S.Snoussi [25]).

The results of this survey will be very useful for the surveillance and the control of abortion diseases.

## Results

### Seroprevalence of abortion causing agents

A total of 793 blood samples were collected from 26 sheep flocks. At the individual level, the overall seroprevalence rate of toxoplasmosis, Q fever, and brucellosis was estimated at 19.7%, 17.2%, and 16.1%, respectively. Toxoplasmosis was the most prevalent abortion disease among the ewes in the organized flocks with a history of abortions (*p* < 0.0001). At the herd level, the number of infected flocks ranged between 19 positive herds with brucellosis and 22 positive herds with toxoplasmosis and Q fever (Table 1).

**Table 1:** The herd and individual seroprevalence rates of the abortion causing agents

Disease	No. of tested herds	No. of positive herds	Seroprevalence rate (%)	No. of tested ewes	No. of positive ewes	Seroprevalence rate (%)
Toxoplasmosis	26	22	84,6%	793	156	19,7% (156/793)
Q fever	26	22	84,6%	793	136	17,2% (136/793)
Brucellosis	26	19	73,1%	793	128	16,1% (128/793)

Our results revealed the presence of mixed infections at the individual and flock levels. At the herd level, 46.1% (12/26) of the investigated flocks presented antibodies against *Brucella* *spp, Toxoplasma gondii* and *Coxiella burnetii.* At the individual level, mixed infections with two and three abortive agents were detected in 9.1% (72/793) and 0.5% (4/793) of the sampled ewes respectively.

### Risk factors

Univariable analysis of risk factors of abortion causing agents (Table 2) showed that the following risk factors. The infection risk was significantly higher in farms where the history of infertility and control of new introduction with the brucellosis and Q fever (*p* < 0.05).The investigation of potential risk factors revealed statistically significant increase in the risk in farms where the exchange of workers between farms and practice the common grazing with the brucellosis and toxoplasmosis (*p* < 0.05). Watering points,and the presence of abortion in neighboring farms, were strongly associated with the toxoplasmosis and Q fever (*p* < 0.05). However, the seroprevalence of these abortion diseases was uniformly distributed among the age group (*p* > 0.05). The difference between the breed of the investigated ewes was statistically significant for the three agents causing abortion. The presence of lambing boxes (for toxoplasmosis) and herd size (for brucellosis) were significantly associated with only one abortion agent (*p* < 0.05). At the individual level, the breed is strongly associated with the seroprevalence of the three abortion diseases (*p* < 0.05).

**Table 2:** Univariable analysis of abortion-related factors of sheep in the organized farms in Tunisia

Risk factors	Category	Brucellosis			Toxoplasmosis			Q fever		
		**No. of negative**	**No. of positive**	***P***	**No. of negative**	**No. of positive**	***P***	**No. of negative**	**No. of positive**	***P***
History of infertility	No	608	105	**0.0012**	569	144	0.2	609	104	**0.0000**
	Yes	57	23		68	12		48	32	
Control of new introduction	No	157	44	**0.01**	157	44	0.3	135	66	**0.0000**
	Yes	508	84		480	112		522	70	
Presence of lambing boxes	No	75	11	0.37	57	29	**0.0005**	74	12	0.4
	Yes	590	117		580	127		583	124	
Common grazing	No	202	82	**0.0000**	192	92	**0.0000**	236	48	0.8
	Yes	463	46		445	64		421	88	
Exchanges of workers between farms	No	633	128	**0.011**	605	156	**0.004**	634	127	0.09
	Yes	32	0		32	0		23	9	
Common watering points	No	514	100	0.83	517	97	**0.0000**	497	117	**0.008**
	Yes	151	28		120	59		160	19	
Presence of abortion on neighboring farms	No	356	72	0.57	360	68	**0.003**	339	89	**0.003**
	Yes	309	56		277	88		318	47	
Age classes (years)	Class I ( ≤ 3 years)	200	40	0.7	195	45	0.6	204	36	0.2
	Class II ( ≥ 4 years)	465	88		442	111		453	100	
Breed	Barbarine	437	90	**0.002**	445	82	**0.0002**	439	88	**0.0008**
	Noir de thibar	196	24		158	62		189	31	
	Queue fine de l’Ouest	32	14		34	12		29	17	
Herd Size	[size < 300]	388	94	**0.0013**	394	88	0.2	392	90	0.1
	[size > 300]	277	34		243	68		265	46	

Of the ten variables, seven, six, and five variables were associated with toxoplasmosis, brucellosis, and Q fever, respectively, and were included in the multivariable analysis for the final regression model. The result is presented in Table 3 and indicated that history of infertility (OR = 4.1 95% CI 2.1- 8.2, *p* = *0.0000*), control of new introduction (OR = 2.05 95% CI 1.14–3.7, *p* = *0.01*) had strong associations with brucellosis. A higher risk of infection with toxoplasmosis is observed among herds that use common watering points (OR = 3.8 95% CI 2.3–6.5, *p* = 0.000). Herd with a history of infertility were more likely to be infected with Q fever (OR: 2.6; 95% CI: 1.4–4.7, *p* = 0.0001). The presence of lambing boxes, breed, common grazing, and flock size was found to be protective factors in the final model for abortion diseases. However, the exchange of workers with farms was not associated with any abortion causing agents (*p* = *0.9*) (Table 3).

**Table 3:** Multivariate logistic regression analysis of risk factors associated with abortion diseases seroprevalence

Risk factor	Category	Brucellosis		Toxoplasmosis		Q fever	
		**OR(95%CI)**	**P**	**OR(95%CI)**	**P**	**OR(95%CI)**	**P**
History of infertility	**Yes**	4.1 (2.1,8.2)	**0.0000**	-	-	2.6 (1.4,4.7)	**0.0001**
	**No**						
Control of new introduction	**Yes**	2.05 (1.14,3.7)	**0.01**	-	-	0.3 (0.2,0.4)	**0.000**
	**No**						
Presence of lambing boxes	**Yes**	-	-	0.55 (0.3,0.9)	**0.04**	-	
	**No**						
Common grazing	**Yes**	0.09 (0.05,0.16)	**0.0000**	0.25 (0.16,0.39)	** < 0.001**	-	
	**No**						
Exchanges of works with other farms	**Yes**	0 (0,Inf)	0.983	0 (0,Inf)	0.982	-	
	**No**						
Common Watering point	**Yes**			3.8 (2.3,6.5)	**0.0000**	0.3 (0.16,0.6)	**0.004**
	**No**						
Presence of abortion in neighboring flocks	**Yes**	-		1.8 (1.2,2.7)	**0.0000**	0.5 (0.3,0.8)	**0.002**
	**No**						
Breed	**Noir de Thibar / Barbarine**	0.24 (0.1,0.4)	**0.0000**	0.7 (0.4, 1.2)	0.2	1.4 (0.7,2.4)	0.746
	**Queue Fine de l’Ouest/ Barbarine**	1.18 (0.5,2.6)	0.6	1.05 (0.4,2.3)	0.9	1.1 (0.4,2.5)	0.7
Flock size	**[size < 300]/[size > 300]**	0.32 (0.19,0.53)	**0.0000**				
						-	

*OR* Odds Ratios and *CI* Confidence Interval

## Discussion

This study provided crucial information on the seroprevalence of three abortive diseases: brucellosis, Q fever, and toxoplasmosis in organized sheep farms with a history of abortion and their potential associated risk factors. The choice of the i-ELISA technique to carry out this investigation is justified by the fact that it is considered to be one of the most sensitive serological techniques (Longbottom et al. [26]).

The limits of this study were essentially related to the category of investigated flocks that were with abortion problems and as a consequence, the seroprevalence of the abortive diseases may not reflect the true seroprevalence of these diseases at the national level.

Our study confirmed the presence of the three abortion causing agents (*Brucella spp*, *Toxoplasma* *gondii,* and *Coxiella burnetii*) in the investigated flocks with high seroprevalence rates ((84.6% for the toxoplasmosis and Q fever). This remarkably high flock-level rate of infection revealed that these abortion diseases are widely spread among the organized sheep flocks. These findings suggest that these two abortion diseases were more prevalent than brucellosis and could be one of the reasons for sheep abortion in Tunisia. At the individual level, the highest seroprevalence was found for toxoplasmosis (19.7%) and the lowest value was attributed to the brucellosis. This result agrees with previous studies in Tunisia that were carried out by Gharbi et al. in [15] in the North of Tunisia, (Gahrbi et al. [15]). Contrary to our result, the seroprevalence of toxoplasmosis found in previous studies in Tunisia (Lahmar et al. [27], Rekiki et al. [7]) was 47%. likewise in Morocco, Ghana, and Roumania seroprevalence rates ranged between 20.8% and 50.6% (Benkirane et al. [28], Hotea et al. 2021 [29], Bentum et al. [30]). Compared to the international figures, a lower seroprevalence rate of toxoplasmosis was reported in Algeria (8.28%), Iran (14.4%), and Turkey (10%) (Dahmani et al., [31], Bahreh et al. [32], Özmutlu et al. [33]).

The level flocks seroprevalence rate of *Coxiella burnetii* was estimated at 84.6% which is higher than the observed rate in the study conducted by Rekiki et al. in 2005 [7] and Khabou et al. in 2009. At the animal level, the infection is less frequent and only 17.2% of positive animals. On the contrary, previous studies in Tunisia revealed high individual seroprevalence rates reported by khbou et al. (2009) and Elandolsi et al., [10] with 32.7% and 56,8%, respectively. Our result is in agreement with the studies conducted on sheep in Algeria, Morocco, and Italy where the seroprevalence of the infection with *Coxiella burnetii* ranged between 14.1% and 15.9% (Khaled et al., [34], Benkirane et al., [28], Rizzo F, et al.[35]). However, the seroprevalence of Q fever in our study is slightly higher than those reported by (Klemmer et al. [36], and Ruiz-Fons et al. [37]).

Regarding *Brucella* spp., this study highlighted high seroprevalence of brucellosis at the flocks level (73%) and low value at the animal level (16.1%) indicating that also brucellosis is widespread among organized flocks in Tunisia. Previous studies have shown that brucellosis was absent among this category of flocks (Rekik et al. [7]), which demonstrates the effectiveness of control that was limited in time. Nevertheless, recent studies are on our results and revealed that the overall animal seropositivity to *Brucella*, was 20.57% in Gafsa district (Khbou et al. 2018). In addition, the high incidence of brucellosis in humans in the latest years (4.35/100,000 inhabitants in 2016 and 9.8/100,000 inhabitants in 2017, confirms that brucellosis is widespread. Our result shows that the epidemiological situation of brucellosis in animals in the field may have changed. Comparatively, the seroprevalence of brucellosis found in this study is higher than reported in Libya (9.2%), Niger (3.6%), and in Algeria (3.8%) (Al-Griw et al. [38], Boukary et al. [39], Zemmouri et al. [40]).

According to our survey, toxoplasmosis was the most prevalent abortion disease with 19.7% of the tested sheep and 22 flocks among the 26 investigated flocks which are in the range of previous surveys in the center of the country (19%) (Gharbi et al. [15]). Similar to the Q fever and brucellosis, this study showed that toxoplasmosis was widely spread among the organized flocks in Tunisia. The overall seroprevalence of the infection with toxoplasmosis observed in this study is lower than reported in the south of Tunisia, Ethiopia Algeria, and Italy (Lahmar et al. [27], Alemayehu et al. [22], Ouchetati et al. [41], Cenci-Goga et al. [42]). However, it is not to the data of previous studies in South Africa and China (Samra et al. [43]).

In this current study, a multi-infection with two or more abortion causing agents was identified at the herd and individual levels. A high percentage of flocks (46.1%) presented antibodies against the three abortion diseases (Brucella, Toxoplasmosis, and Q fever). At the individual level, mixed infections with two and three abortive agents were also detected in sampled ewes. In agreement with the present findings, the association between different abortifacient agents was reported by Rekik et al. [7] where an association of two to five abortion infections was detected in the investigated flocks. The presence of mixed infection with abortion causing agents is not unique but rather common in many regions. The results from the surveys conducted in Morocco revealed that the majority of herds presented several potentially abortive infections (2 to 5 abortifacient agents) (Benkirane et al., 2005 [28]), and highlighted the high rate of mixed infection (67%) at the flocks level (El Idrissi A.H &all. [44]). In Greece, Bisias et al. [45] reported a similar finding. It is important to point out that in mixed infection it is extremely difficult to identify the actual agent responsible for the abortion. Indeed, several authors claim that the responsible infectious agent is only diagnosed in 30—50% of cases (Radostis et al., [46], Corbellini et al., [47], Kim et al., [48]).

Our study highlighted the multifactorial nature of the brucellosis, Q fever, and toxoplasmosis infection in the organized flocks and also emphasize the potential role of the management practices as control of new introduction, common grazing and watering, and the presence of lambing boxes in the farms in the increase of the infection risk with the causing abortion agents. Regarding the history of infertility as a risk factor for the three abortive diseases, our result showed that flocks with problems of infertility had 4.1 (OR = 4.1, 95% CI = 2.1–8.2) and 2.6 (OR = 2.6, 95% CI = 1.4–4.7) times higher risk for infection with brucellosis and Q fever, respectively. This is not surprising since the ewes with reproductive problems were more exposed to abortion and an association with infertility and abortive diseases was reported (Martini et al. [49], Ullah et al. [50], Sorsa M et al. [51]). Control of new introduction is significantly associated with the seroprevalence of brucellosis and farms practicing the introduction of animals without any test are 2.05 times more likely to be infected. This demonstrates that introduced animals that are quarantined must be tested for brucellosis and the quarantine alone is not sufficient to control this disease since it can be transmitted by semen. Our result is in agreement with the previous studies that demonstrated high seroprevalence of brucellosis in flocks that bought outside animals compared to those that did not (Santos et al. [52], Jimale A.C.A [53]).

The odds of testing positive for toxoplasmosis were 3.8 times higher in the flocks that practice the common watering point. This can be explained by the fact that horizontal transmission of *T. gondii* to sheep by water is considered the most important route of infection in sheep (Stelzer et al. [54]) and the oocytes of the parasite present in the cat feces can contaminate water (Hotea et al. [29]). On the contrary, no statistically significant association was found between toxoplasmosis and common watering point (Abdallah et al. [55], Ahmad et al. [56]). We found that the presence of abortion on neighboring flocks increases by 1.8 times the risk of infection with toxoplasmosis. It was reported that the spread of toxoplasmosis via the oocytes is facilitated by wind, earthworms, and arthropods, as well as by rain (Shapiro et al. [57]). The current study revealed the protective role of the presence of the lambing box for the infection with toxoplasmosis. This demonstrates that the management practice can reduce the prevalence of abortion diseases.

Flock size was found to be significantly associated with the seroprevalence of brucellosis, the number of seropositive animals increases with the decrease of the size of the flock. This finding is in disagreement with previous studies (Boukary et al. [39], Alhamada et al. [58]). The multivariable model analysis identified the breed Barbarine as a protective factor for brucellosis seropositivity and this could be related to the resistance of this breed (Atti et al. [59]).

The seroprevalence figures described in this study demonstrate that the three abortion diseases are widely distributed across organized sheep flocks in Tunisia. The multivariate model highlights the importance of biosecurity, the management practice, and the individual factors in the risk of the infection causing abortion agents. A study that targets all Tunisian herds is recommended to confirm our results and estimate the true seroprevalence of these diseases.

## Conclusion

This study revealed the widespread abortion diseases within the organized sheep flocks in Tunisia. Despite the serological diagnosis of infectious abortions is commonly used in veterinary medicine, it is still difficult to interpret and establish a definitive diagnosis for the circulating pathogens. Indeed, to confirm the serological results, the use of direct diagnosis such as bacteriological isolation and identification or PCR tests is indispensable. The detection and the identification of pathogens are essential steps for the control and management of these diseases. In this study, the co-infection was demonstrated and identified, these findings were very useful to adapt and adjust the control and response plan to reduce the risk of the spread of these infections among flocks. Further investigations are needed to improve knowledge, assess the extent of the problem, and therefore develop more effective control strategies to reduce these abortive zoonoses., therefore, the adoption of hygiene and biosecurity practices is recommended as the transmission between animals, wildlife, and humans is a “One Health” approach.

## Methods

### Study area

**Fig. 1:**
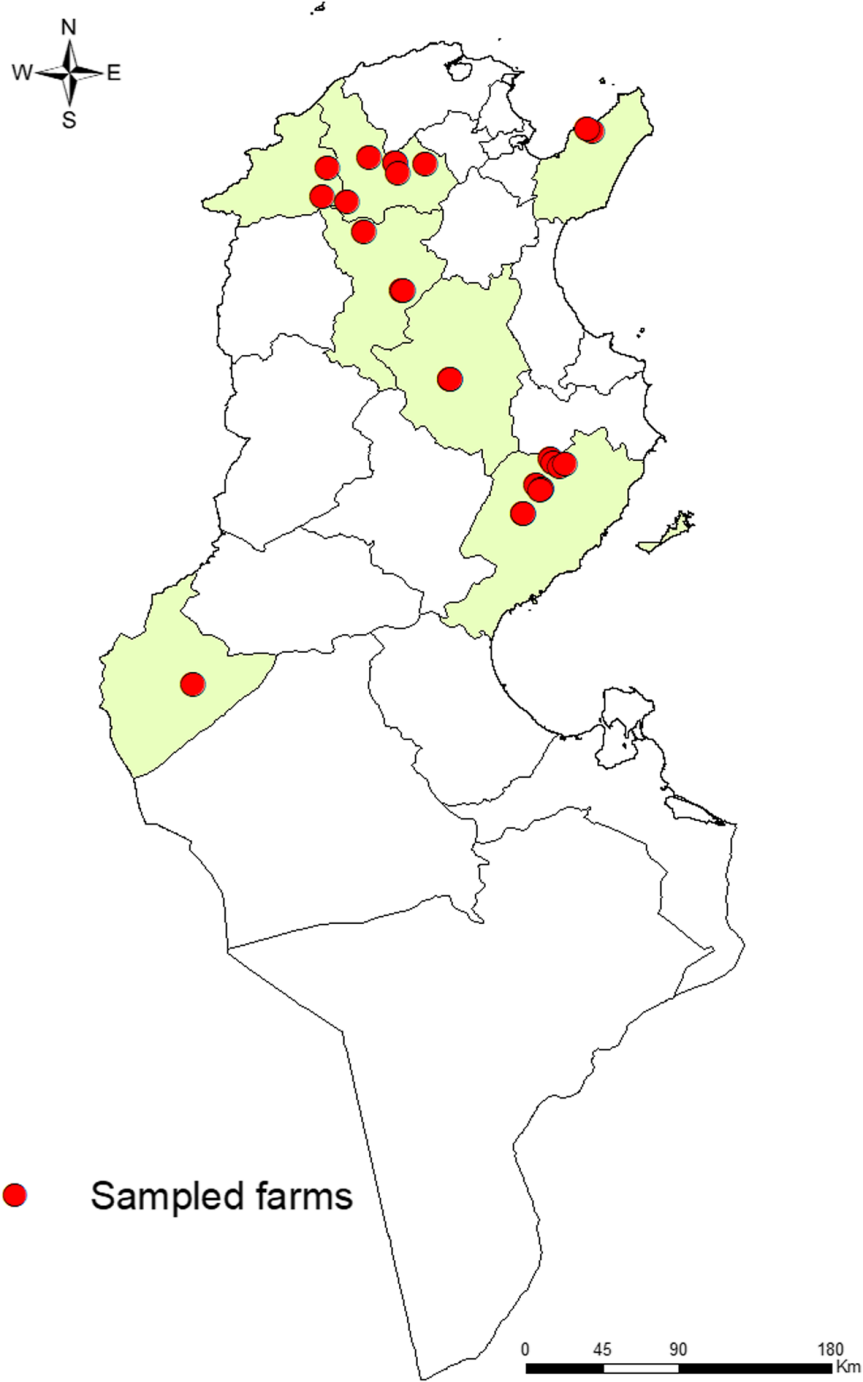
Map of Tunisia showed the locations of sampled sheep flocks

The study was performed in seven governorates from the north (Governorates of Jendouba, Beja, Siliana, and Nabeul), the Centre (Governorates of Kairouan and Sfax), and the south of Tunisia (Governorate of Tozeur). These governorates were divided into 76 districts and 744 sectors, covering an area of 34.087 km^2^ and a total population of 3,513,537 inhabitants (Fig. 1). The mean annual rainfall and the annual mean temperature of the study area ranged from 50 to 1000 mm and from 19 to over 25 °C, respectively.

### Study design and sample size determination

The target population is represented by 132 organized sheep farms where the number of sheep ranged from 130 to 450 ewes. A cross-sectional study was conducted between September 2014 and May 2018 with sheep from a sample of organized farms with a history of abortion identified in a previous survey conducted in 2015. As a result, 26 organized farms out of 132 farms with 6501 females, considered as herds with “abortion problems” were sampled. The sample size was calculated with EpiTools (Sergeant [60]) using the following assumptions:

- An expected prevalence of 10% was considered for the three pathogens based on the literature review.
- A desired absolute precision of 20%
- Risk of error of 5%.

Seven hundred sixty-four females were required and randomly selected for the survey. To prevent missing data, several additional samples were programmed (*n* = 29) which represent 3.6% of the total samples. At the herd level, ewes were randomly selected using the identification database with the proportional allocation of the number of samples to the herd size.

### Data collection

A detailed and structured questionnaire was used to collect information about the flocks (size, GPS coordinates), animals (age, breed, stage of abortion), and risks factors related to abortion diseases (presence of lambing boxes, history of infertility, control of new introduction, quarantine, common watering points, and grazing areas, exchanges of workers between farms, presence of abortion in neighboring farms).

#### Blood samples collection

Whole blood was collected from the jugular vein of each animal using one sterile Vacutainer tube (5 ml), without anticoagulant for the serological test. Blood samples were immediately cooled to 4 °C and sent to the laboratory. Serum samples were harvested by centrifugation at 3,000 × g for 10 min then stored in labeled Eppendorf tubes at − 20 °C until testing, as recommended by the OIE (OIE [61]).

#### Laboratory analysis

The serum samples were tested by indirect enzyme-linked immunosorbent assay (i-ELISA), using a multi-species test kit, according to the manufacturer's instructions (ID Screen® Q fever, ID Screen® toxoplasmosis, and ID Screen® Brucellosis Serum Indirect Multi-species, IDVET Innovative Diagnostics™, Montpellier, France.

Sera samples were considered positive where antibodies against T. gondii is > 40%, for Q fever: if the antibody titer is ≥ 40% and ≤ 80%. It is qualified as "strongly positive" if this titer is > 80%, and for Brucellosis if the antibody titer is ≥ 110,

The sensitivity and specificity of the i-ELISA test were estimated to be 100% and 99.6%, respectively, according to the manufacturer. A herd is classed as positive if at least one animal is seropositive to one of the three abortion causing agents.

#### Statistical analysis

Collected data were entered into a database using Microsoft Access. The overall animal-level prevalence was estimated as the ratio of the number of positive animals to the total of tested samples. The flock-level seroprevalence was calculated by dividing positive flocks by the total number of flocks tested.

History of infertility, control of new introduction, presence of lambing boxes, common grazing areas and watering points, exchanges of workers between farms, presence of abortion in neighboring farms, age group, herd size, and breed are exposure variables were considered for the univariate and multivariate analysis.

The associations between the different risk factors and the presence of antibodies against abortion agents was performed by using Pearson’s chi-square test. Risk factors showing a significance level α at a P ≤ 0.05 were retained to construct the final multivariate logistic regression model. The significance level α was set at a P-value ≤ 0.05. Analyses were performed using R software (R Core Team, [62]; version 3.5.3). Maps were generated with ArcGIS version 10.4 (https://www.esri.com/). The Hosmer and Lemeshow χ2 test was performed to evaluate the goodness of fit. The area under the curve (AUC) of the Receiver Operating Characteristic (ROC) was plotted for the three abortion causing agents to estimate the predictive ability of the three models.

## Data Availability

The raw data generated and analyzed during the current study are not publicly available due to restrictions in publishing data relevant which is confidential and proprietary to the institution. Data are available from the corresponding author on reasonable request.
